# Leucine-rich-repeat-containing variable lymphocyte receptors as modules to target plant-expressed proteins

**DOI:** 10.1186/s13007-017-0180-8

**Published:** 2017-04-19

**Authors:** André C. Velásquez, Kinya Nomura, Max D. Cooper, Brantley R. Herrin, Sheng Yang He

**Affiliations:** 1 DOE Plant Research Laboratory, Michigan State University, East Lansing, MI 48824 USA; 2 Department of Pathology and Laboratory Medicine, Emory University, Atlanta, GA 30322 USA; 3 Department of Plant Biology, Michigan State University, East Lansing, MI 48824 USA; 4 Plant Resilience Institute, Michigan State University, East Lansing, MI 48824 USA; 5 Howard Hughes Medical Institute, Gordon and Betty Moore Foundation, Michigan State University, East Lansing, MI 48824 USA

**Keywords:** Protein targeting, Leucine-rich repeat, Variable lymphocyte receptor, Modules, HopM1

## Abstract

**Background:**

The ability to target and manipulate protein-based cellular processes would accelerate plant research; yet, the technology to specifically and selectively target plant-expressed proteins is still in its infancy. Leucine-rich repeats (LRRs) are ubiquitously present protein domains involved in mediating protein–protein interactions. LRRs confer the binding specificity to the highly diverse variable lymphocyte receptor (VLR) antibodies (including VLRA, VLRB and VLRC types) that jawless vertebrates make as the functional equivalents of jawed vertebrate immunoglobulin-based antibodies.

**Results:**

In this study, VLRBs targeting an effector protein from a plant pathogen, HopM1, were developed by immunizing lampreys and using yeast surface display to select for high-affinity VLRBs. HopM1-specific VLRBs (VLR_M1_) were expressed *in planta* in the cytosol, the *trans*-Golgi network, and the apoplast. Expression of VLR_M1_ was higher when the protein localized to an oxidizing environment that would favor disulfide bridge formation (when VLR_M1_ was not localized to the cytoplasm), as disulfide bonds are necessary for proper VLR folding. VLR_M1_ specifically interacted *in planta* with HopM1 but not with an unrelated bacterial effector protein while HopM1 failed to interact with a non-specific VLRB.

**Conclusions:**

In the future, VLRs may be used as flexible modules to bind proteins or carbohydrates of interest *in planta*, with broad possibilities for their use by binding directly to their targets and inhibiting their action, or by creating chimeric proteins with new specificities in which endogenous LRR domains are replaced by those present in VLRs.

**Electronic supplementary material:**

The online version of this article (doi:10.1186/s13007-017-0180-8) contains supplementary material, which is available to authorized users.

## Background

In order to relay signals and interact with other molecules, proteins have acquired certain commonly used repetitive domains. One domain that has been shown to be involved in mediating protein–protein interactions is the leucine-rich repeat (LRR) domain, which is present in a variety of proteins from all domains of life including bacteria, eukaryotes, and even viruses [1]. Each LRR domain contains a conserved segment with the consensus sequence LxxLxLxxN/CxL (where L, C, and N stand for leucine, cysteine, and asparagine, respectively, while x stands for any amino acid) and adopts the secondary structures of a β strand and an α helix connected by a loop [2]. Multiple LRR domains arranged in tandem form a crescent-shaped structure, in which a continuous β-sheet on the concave side forms the most common surface for protein–protein interactions [1]. The versatility of LRR domains in mediating protein–protein interactions is exemplified by the vast functions that proteins containing this domain may fulfill. For example, in plants, LRR domains are present in transmembrane receptor proteins of the LRR-receptor kinase (LRR-RK) and LRR-receptor protein (LRR-RP) classes [3–6], in intracellular nucleotide-binding site–LRR proteins (NBS–LRR) [7], in F-box proteins including hormone receptors of the E3 ubiquitin ligase class [8, 9], in a component of nucleocytoplasmic transport (RanGAP) [10], in intracellular proteins involved in pollen development (PIRLs) [11], and in extracellular cell wall LRR-extensins [12] and polygalacturonase-inhibiting proteins (PGIP) [13]. The first three classes have radiated exponentially in plants and account for almost 3% of all genes in Arabidopsis [14, 15], and have been shown to be primarily involved in defense signaling and recognition, and plant development.

Variable lymphocyte receptors (VLRs) are non-self recognition receptors present in jawless vertebrates (Agnatha, which includes hagfishes and lampreys), involved in detecting invading microbes. They are the functional equivalent of immunoglobulin-based antigen receptors and antibodies in jawed vertebrates (Gnathostomata, which includes all other vertebrates from cartilaginous fish to mammals) [16–19]. Contrary to the immunoglobulin domains used by gnathostomes, VLR antibodies primarily bind to antigens using the concave surface formed by their LRR domains [20]. VLR proteins have the following domains: a signal peptide, an N-terminal LRR (LRRNT), multiple LRRs with variable sequence (up to 10 have been observed in a mature VLR [21]; the first LRR and the last one are referred as LRR1 and LRRVe, respectively), an incomplete LRR (connecting peptide, LRRCP), a C-terminal LRR (LRRCT), and a flexible, invariant stalk followed by a transmembrane or glycosylphosphatidylinositol (GPI)-anchor region [21]. Both the N- and C-terminal LRR domains have two characteristic disulfide bridges to stabilize the fold of the protein [20]. Agnathans possess T-like and B-like lymphocytes in which each differentiated lymphocyte carries a unique set of variable LRR sequences in their mature *VLR* gene [22]. The high variability in the LRR region of VLRs has been estimated to allow a potential repertoire of 10^14^–10^17^ VLR variants, a feat that is achieved by somatic diversification through the step-wise incorporation of different LRR donor sequences into the incomplete germline gene until an in-frame functional mature VLR is formed [23].

Three different VLRs exist in lampreys and hagfishes; VLRA, VLRB, and VLRC; with individual lymphocyte lineages only expressing a single functional VLR type [22, 24]. *VLRA* and *VLRC* are expressed by lymphocytes that resemble jawed vertebrate T cells. After antigen stimulation, these T-like lymphocytes proliferate and increase expression of proinflammatory cytokines, while their antigen receptors always remain attached to the cell surface [22, 25]. In contrast, *VLRB*-expressing lymphocytes differentiate into plasmablasts that secrete their VLRB receptors as disulfide-linked multimers that serve as the functional equivalent of jawed vertebrate antibodies [26, 27].

In this study, the feasibility of using LRR-containing lamprey-derived VLRBs to target *in planta*-expressed proteins was investigated. The VLRBs were shown to accumulate in different cellular compartments, and VLRBs that were targeted through the plant secretory pathway were indeed able to interact *in planta* with their target, HopM1, a bacterial effector protein from a plant pathogen. These results provide a proof-of-concept demonstration for engineering VLR-based protein-targeting LRR modules *in planta*.

## Results

**Fig. 1:**
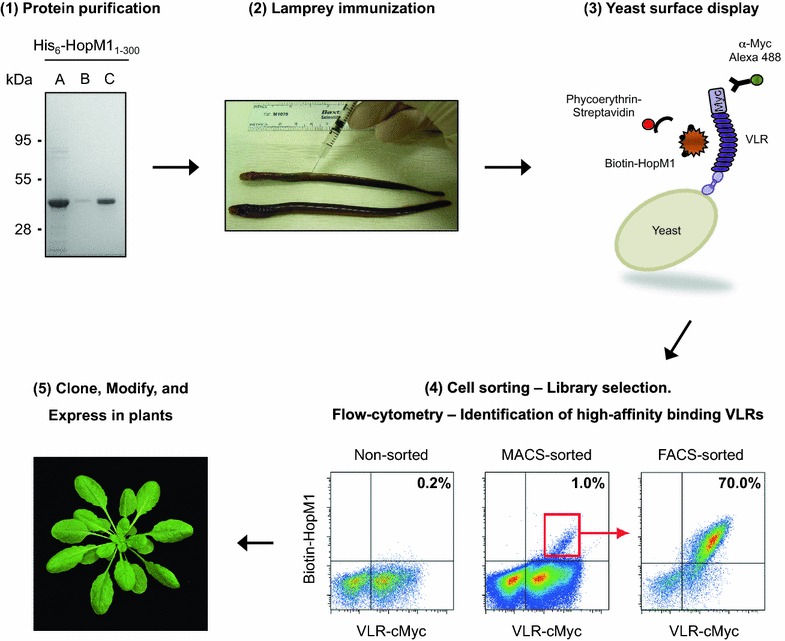
Variable lymphocyte receptors as tools to target plant-expressed proteins. A schematic diagram depicting the steps involved in developing an LRR-containing VLR that binds to plant-expressed proteins. (*1*) Express and purify the protein from *E. coli*, *P. pastoris*, or other sources. (*2*) Immunize lampreys with the purified protein of interest conjugated to an adjuvant for the production of VLRB antibodies. (*3*) Clone VLRBs from lamprey’s lymphocytes into a yeast surface display (YSD) library. (*4*) Enrich the YSD library for high-affinity binding VLRBs using magnetic-activated cell sorting (MACS) and fluorescence-activated cell sorting (FACS), and identify individual high-affinity binding VLRBs using flow cytometry. (*5*) Clone VLRBs into plant expression vectors for *in planta* expression. The LRR-containing VLR may be modified to carry additional modules (e.g., enzymes or receptors). Step 1 shows Denville Blue™ staining of SDS-PAGE gel of *E. coli* expressed His_6_-HopM1_1–300_. (*A*) Ni–NTA agarose purified protein. (*B*) Anion-exchange chromatographic flow-through. (*C*) Fraction eluted with 433 mM NaCl from the anion exchange chromatographic column, which after dialysis into phosphate buffer was used to inoculate lampreys for VLRB production. Step 4 shows the YSD library before enrichment for VLRBs that bind HopM1_1–300_ with high affinity (non-sorted), and after MACS and FACS selection

The methodology developed for producing VLR-based LRR modules for targeting plant-expressed proteins starts by expressing and purifying the protein of interest, typically using at least two chromatographic purification steps to have a high-purity target protein, so that non-specific VLRs against contaminants are not also produced (Fig. 1). The purified protein is conjugated to an adjuvant (mammalian Jurkat T cells), since in lampreys, soluble proteins are weakly immunogenic on their own. The conjugated target protein is injected into lampreys for inducing the production of VLRB antibodies and, after confirming using ELISA that VLRB antibodies are present in the plasma of immunized lampreys, a yeast surface display (YSD) library is prepared by cloning the lymphocyte *VLRB* transcripts. The cloned *VLRB*s lack the N-terminal signal peptide and C-terminal anchor regions, and are fused to yeast protein Aga2p, so that they become attached to the cell wall of yeast cells after secretion [28]. VLRBs are also fused to a C-terminal c-Myc epitope for VLRB detection during yeast surface display. VLRB binding to the antigen of interest on the surface of yeast cells is detected by flow cytometry using a biotinylated antigen and fluorescently labeled streptavidin.

Typically, only 0.1–0.5% of VLRBs in the immune-stimulated YSD library have sufficient affinity for detection of antigen binding by flow cytometry. Therefore, the YSD library is enriched for antigen-specific high-affinity VLRBs using one or two rounds of magnetic-activated cell sorting (MACS) with streptavidin-conjugated magnetic beads before using fluorescence-activated cell sorting (FACS) to specifically isolate the highest affinity clones. The FACS-sorted yeast cells are plated and individual yeast clones are tested for antigen binding. The nucleotide sequence of the *VLRBs* from high-affinity antigen-binding clones is determined and the *VLRBs* are cloned into plant expression vectors. Transient expression or stable transformants are then generated through *Agrobacterium*-mediated transformation of plants, after which the *in planta* binding of the VLRB to the antigen of interest and any phenotypes of interest can be evaluated.

### Development of VLRBs against the bacterial effector HopM1

HopM1 is an effector from *Pseudomonas syringae* encoded in the conserved effector locus (*CEL*) [29]. HopM1 is not only one of the most conserved effectors in *P. syringae* strains [30], but also its *in planta* localization and target are known [31, 32]. We decided to test the feasibility of using LRR-containing VLRBs *in planta* to target HopM1. The N-terminus of HopM1 (amino acids 1–300; HopM1_1–300_) fused to an N-terminal hexahistidine tag was expressed and purified from *Escherichia coli* (Fig. 1). HopM1_1–300_ was used instead of full-length HopM1 because of increased protein solubility and ease of purification. Purification was performed by using Ni–NTA agarose beads and ion-exchange chromatography. Purified N-terminal HopM1 was covalently conjugated to paraformaldehyde-fixed Jurkat T cells (as an adjuvant) and used to inject lamprey larvae to induce production of VLRB antibodies against HopM1 (VLR_M1_). Three lampreys were immunized a total of three times at 2-week intervals. After the final immunization, blood plasma was collected from the lampreys and tested for binding to HopM1_1–300_ by ELISA. Plasma from lamprey-1 had the highest binding to HopM1_1–300_ (at almost a 1 in a 1000 dilution of the plasma; Additional file 1: Figure S1), and as such, the *VLRB* repertoire from this lamprey was PCR amplified from total lymphocyte cDNA and used to construct a YSD library (of approximately 1.1 × 10^6^ clones) to select for VLR_M1_ clones. The YSD library was enriched for clones with high-binding affinity for HopM1 by one round of MACS sorting using 100 nM of biotinylated HopM1_1–300_, before FACS sorting for yeast cells expressing higher affinity VLR_M1_ clones were selected (Fig. 1).

**Fig. 2:**
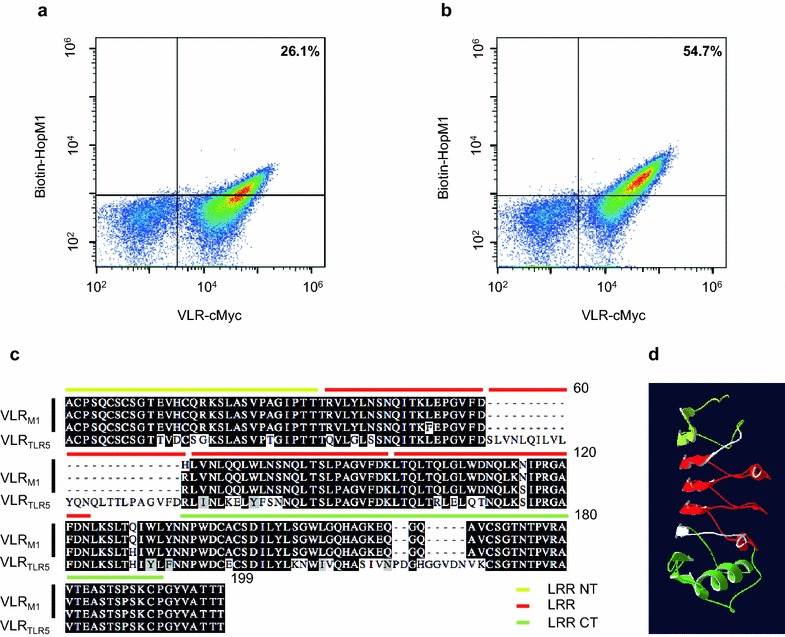
Identification of variable lymphocyte receptors that bind the *Pseudomonas syringae* effector HopM1. **a** and **b** yeast-surface display of HopM1-specific VLRBs. The *x-axis* represents Alexa Fluor^®^ 488 (conjugated to α-c-Myc antibody) fluorescence while the *y-axis* shows phycoerythrin (conjugated to streptavidin) fluorescence of individual yeast cells. Fluorescence was detected using BD Accuri C6 flow cytometer. The number highlighted in *bold* indicates the percentage of yeast cells with detectable HopM1_1–300_ binding. **a** Lower-affinity binding HopM1-specific VLRB; 50 nM biotinylated HopM1. **b** Higher-affinity binding HopM1-specific VLRB; 50 nM biotinylated HopM1. **c** Amino acid alignment of the three different high-affinity HopM1-specific VLRB sequences and of Toll-like receptor 5 (TLR5; a mammalian immune receptor that recognizes bacterial flagellin)-specific VLRB. Alignment was generated using MegAlign (DNASTAR) and graphed using Boxshade. Highlighted in a *white background* are amino acids that are different. Amino acid position is shown on the upper right corner. A *red bar* over the sequence identifies the leucine-rich repeat (LRR) domains identified. In *yellow* and *green*, are the N-terminal and C-terminal LRR domains, respectively, which are characterized by the presence of disulfide bonds. The domains were identified using the SMART tool [59]. **d** 3-D structure model of HopM1-specific VLRB. LRR domains are highlighted in *red*, the N-terminal LRR domain in *yellow*, and the C-terminal LRR domain in *green*. Modeling was performed with SWISS-MODEL using the structure of a previously crystalized VLRB protein (3g3aA) [16]

Forty randomly selected VLR_M1_-expressing yeast colonies from the FACS-sorted library were individually tested for binding to HopM1. The strengths of binding varied among these clones (Fig. 2a, b). The *VLRB* gene from nine colonies with the highest binding affinity to HopM1 was sequenced. All nine *VLRB* clones carried a strikingly similar sequence in which less than 2% of nucleotides were polymorphic, which translated into only 4 amino acids (out of 168; 2.4%) being different (Fig. 2c). VLR_M1_ carried 3 LRRs (LRR1; LRRV, for LRR variable; and LRRVe) flanked by N-terminal and C-terminal LRRs. This number of LRR domains is very close to the average number of LRRs, 3.81, observed in VLRBs [20]. We performed homology modeling of the structure of VLR_M1_ (the uppermost VLR_M1_ sequence from Fig. 2c was used for this analysis and for the remainder of the experiments, unless indicated otherwise) using a lysozyme-specific VLRB (VLR_HEL_) [16]. This analysis revealed the characteristic structure for VLRBs, a solenoid forming an arc, in which the β-strands in the concave surface (with the sequence xxLxLxx, in which L stands for leucine and x for any amino acid) are predicted to be involved in the binding interaction with HopM1 (Fig. 2d).

### *In planta* expression and visualization of VLR_M1_

**Fig. 3:**
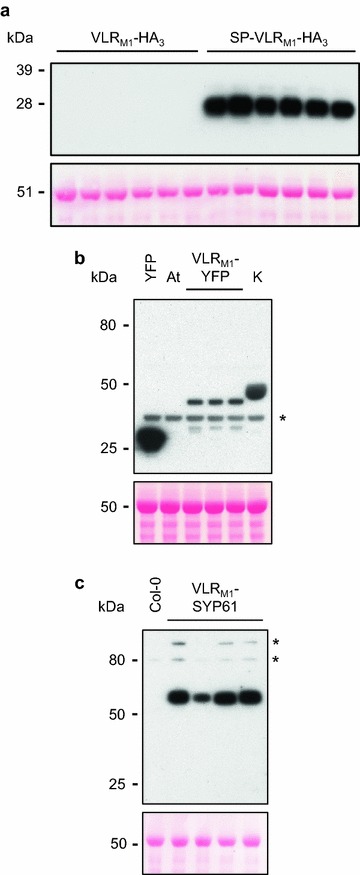
*In planta* accumulation of VLR_M1_ is higher if the protein goes through the secretory pathway. **a** Western blot of cytoplasmic and apoplastic VLRBs fused at their C-terminus with three HA tags detected with α-HA antibodies. Eight µg of total protein were loaded per well. Expression of three different high-affinity HopM1-specific VLRBs (done in duplicate) with slightly different amino acid sequences (see Fig. 2c) was performed for the cytoplasmic (VLR_M1_-HA_3_) and apoplastic (SP-VLR_M1_-HA_3_) versions of the HopM1-specific VLRB. A Ponceau S staining of the membrane is shown below the blot to confirm similar sample loading of the gel. Accumulation of only SP-VLR_M1_ was observed. *SP* signal peptide. **b** Western blot of cytoplasmic VLRB fused at its C-terminus with YFP detected with α-GFP antibodies. Twenty-nine µg of total protein were loaded per sample. Expression of three different high-affinity HopM1-specific VLRBs with slightly different amino acid sequences was performed. YFP and HopK1-YFP (K) were used as positive controls, while an *A. tumefaciens* strain (At) devoid of any plant expression vectors was used as a negative control. The *asterisk* represents the position of a non-specific band. A Ponceau S staining of the membrane is shown below the blot to confirm similar sample loading of the gel. **c** Western blot of cytoplasmic VLRB fused at its C-terminus with syntaxin SYP61 (*At1g28490*) and three HA tags detected with α-HA antibodies. Twenty-three µg of total protein were loaded per well. Proteins were extracted from four T_1_ transgenic *A. thaliana* Col-0 plants and an untransformed Col-0 plant. Asterisks represent the position of non-specific bands. A Ponceau S staining of the membrane is shown below the blot to confirm similar sample loading

*VLR*_*M1*_ was expressed in plants under the control of the cauliflower mosaic virus (CaMV) 35S promoter. No accumulation of cytoplasmic VLR_M1_ was observed for any of the three HopM1 high-affinity sequences expressed (Fig. 3a). However, accumulation was detected when VLR_M1_ was fused to YFP, albeit at a low level (Fig. 3b; compare to expression to an unrelated effector from *P. syringae*, HopK1). Since disulfide bond formation in plants occurs in the endoplasmic reticulum (ER) or at the cell wall (except for mitochondrial and chloroplast proteins) [33], and VLRBs have 4 intramolecular disulfide bonds necessary for proper protein folding [20], we decided to express *VLR*_*M1*_ fused to a signal peptide (from *AtPR1*; *At2g14610*), so that the protein would be targeted to the plant secretory pathway. Contrary to cytoplasmic VLR_M1_ accumulation, SP-VLR_M1_ accumulation was readily detectable (Fig. 3a). We also evaluated if targeting VLR_M1_ to a specific cell compartment without utilizing the secretory pathway would increase protein accumulation. Indeed, fusion of VLR_M1_ to syntaxin SYP61, a tail-anchored protein involved in vesicle selection and fusion localized to the early endosome/*trans*-Golgi network (TGN) [34], increased VLR_M1_ accumulation (Fig. 3c). As a tail-anchored protein, SYP61 is inserted post-translationally into the membrane through its hydrophobic C-terminus [35]. If required, VLR_M1_ could be targeted towards the lumen of the TGN, by simply fusing SYP61 to the N-terminus of the protein [36], instead of the C-terminus as was done in this study.

**Fig. 4:**
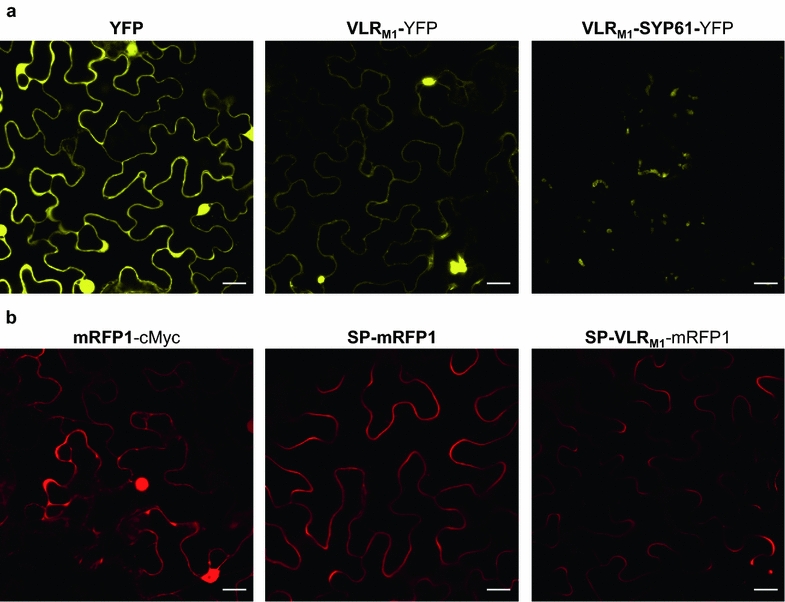
Visualization of *in planta* VLRB protein expression. **a** Expression of intracellular YFP, HopM1-specific VLRB (VLR_M1_), and VLR_M1_ fused to *A. thaliana* syntaxin SYP61 (*At1g28490*) in *Nicotiana benthamiana*. Images were taken with the Olympus FluoView 1000 confocal microscope using a 515 nm laser for YFP excitation, while emission was collected between 530 and 569 nm. Fusing VLR_M1_ to SYP61 targets the VLRB to intracellular compartments, most likely the *trans*-Golgi network. **b** Expression of intracellular mRFP1, and predicted extracellular SP-mRFP1 and SP-VLR_M1_-mRFP1 in *N. benthamiana*. mRFP1 and HopM1-specific VLRB were targeted to the apoplast by fusing the VLRB to the signal peptide (SP) of *Arabidopsis thaliana* PR1 (*At2g14610*). Accumulation on the periphery of the cells was observed. Images were taken with the Olympus FluoView 1000 confocal microscope using a 559 nm laser for excitation and collecting the emission between 570 and 600 nm. *White bar length* represents 20 µm. Image brightness increased 40%

Visualization of fluorescently labeled VLR_M1_ (VLR_M1_-YFP) revealed that the protein localized to the cytoplasm and to large aggregates that did not seem to correspond to the nucleus (Fig. 4a). These aggregates might reflect the accumulation of unfolded VLR_M1_ proteins, as the cytoplasm is not conducive for disulfide bond formation [33]. When VLR_M1_ was targeted to the TGN (VLR_M1_-SYP61-YFP), a punctuate pattern was observed instead (Fig. 4a). The same pattern was observed for SYP61, as had been observed before in transgenic Arabidopsis plants [37] (Additional file 1: Figure S2). To visualize secreted VLR_M1_ in plant cells, SP-VLR_M1_ was fused to mRFP1, as mRFP1 is mostly insensitive to pH changes in the physiological range [38] while YFP fluorescence is quenched at acidic pH (i.e., in the apoplast), and as such, GFP and its derivatives may not be used as fluorescent tags for extracellular proteins. SP-VLR_M1_ was found to be localized to the periphery of the cell with a similar localization to that observed for secreted mRFP1 (SP-mRFP1) (Fig. 4b), and different from that of cytoplasmic and nuclear localized mRFP1.

### *In planta* interaction between VLR_M1_ and HopM1

**Fig. 5:**
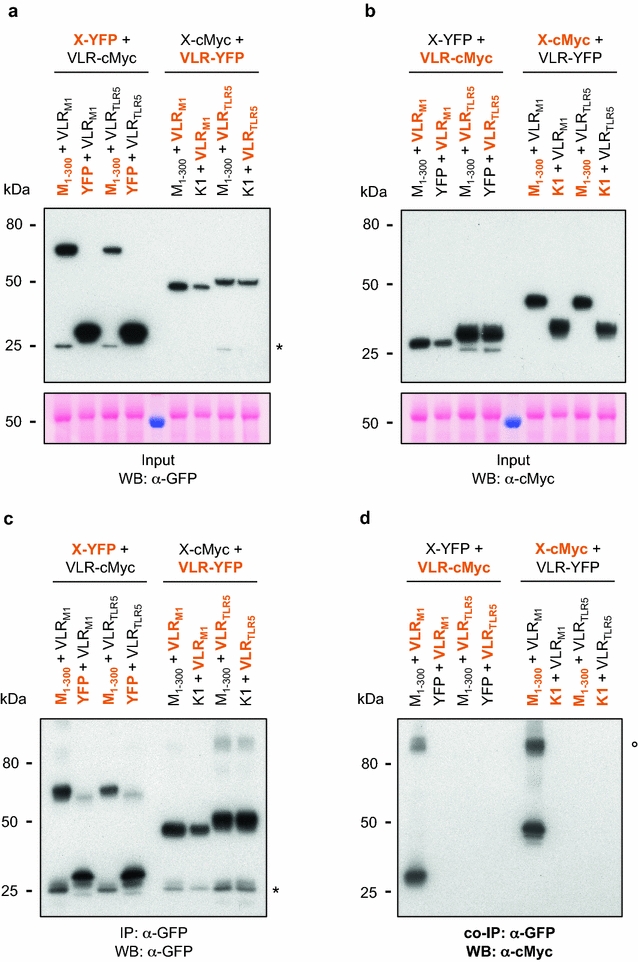
*In planta* interaction of HopM1 with VLR_M1_. Co-Immunoprecipitation of HopM1 and its corresponding VLRB in *Nicotiana benthamiana*. Thirteen and one-half mg of proteins were immunoprecipitated (IP) with α-GFP agarose beads without the use of reducing agents in the buffers. Proteins were detected with either α-GFP (IP) or α-c-Myc (co-IP) antibodies. Interactions between HopM1 and HopM1-specific VLRB were tested with both proteins fused to two different epitope tags. *Highlighted in orange* are those proteins detected in the Western blot, while in *black* are those proteins also expressed but not detected. VLR_M1_ = SP-VLR_M1_, VLR_TLR5_ = SP-VLR_TLR5_, M_1–300_ = SP-HopM1_1–300_, K1 = HopK1. **a** Total protein input of YFP-tagged proteins for IP, Western blot used α-GFP antibodies for protein detection. Ponceau S staining of the input PVDF membrane is shown below the Western blot. The *asterisk* represents the position of YFP cleaved from the fusion protein. **b** Total protein input of c-Myc-tagged proteins for IP. Western blot used α-c-Myc antibodies for protein detection. Ponceau S staining of the input PVDF membrane is shown below the Western blot. **c** IP of YFP-tagged proteins with α-GFP agarose beads. Western blot used α-GFP antibodies for protein detection. The *asterisk* indicates the position of YFP cleaved from the fusion protein. **d** Co-IP of c-Myc-tagged proteins with α-GFP agarose beads. Western blot used α-c-Myc antibodies for protein detection. VLR_M1_ only interacted with HopM1. The *open circle marks* the position of a probable dimer formed between VLR_M1_ and HopM1_1–300_

We next evaluated the feasibility of *in planta* interaction between VLR_M1_ and HopM1 through co-immunoprecipitation experiments. We used apoplast-localized SP-VLR_M1_ for these experiments, as its expression was higher than that observed for cytoplasmic VLR_M1_ (Fig. 3a). As a negative control, we used a randomly selected VLRB sequence from a YSD library that was prepared from lampreys immunized against Toll-like receptor 5 (TLR5, which recognizes bacterial flagellin) [39] (Fig. 2c, note that this VLRB carries 4 LRR domains instead of the 3 observed for VLR_M1_). For the co-immunoprecipitation experiments, a signal peptide for protein secretion was fused to the N-terminus of HopM1 (HopM1_1–300_), so that both HopM1_1–300_ and VLR_M1_ would be localized to the same compartment. As negative controls, we used free YFP and HopK1, the latter of which is a bacterial effector that does not share sequence similarity with HopM1. All proteins (except for YFP and HopK1) were tagged with either YFP or four c-Myc tags so that reciprocal co-immunoprecipitations could be performed. Even though expression of each protein was variable (Fig. 5a, b), the amount of the YFP-tagged proteins immunoprecipitated was equivalent between all samples (Fig. 5c). Specific interaction between HopM1-specifc VLRB and HopM1_1–300_ was clearly observed (Fig. 5d; Additional file 1: Figure S3). No interaction was observed with the unrelated VLRB recognizing TLR5, nor against HopK1 or YFP.

It is important to note that the immunoprecipitations did not use any reducing agents, as attempts to perform the immunoprecipitations with dithiothreitol in the buffer failed, probably because of the importance of the disulfide bonds for proper VLR folding. In the co-immunoprecipitation experiments, a protein band of approximately 100 kDa was observed in the western blot (Fig. 5d; the lithium dodecyl sulfate (LDS) buffer in which immunoprecipitated proteins were resuspended did not contain reducing agents either). The expected molecular weight of a dimer between VLR_M1_ and HopM1_1–300_ is about 90–93 kDa (depending on the tags used), which is very close to the molecular weight of this specific band (protein multimers have been observed before even in denaturing SDS-PAGE conditions [40]), providing further support for the *in planta* interaction between these two proteins.

## Discussion

We have described an original method for targeting plant-expressed proteins using LRR-containing VLRBs (Fig. 1). After lamprey immunization with the protein of interest, yeast surface display is used to identify high-affinity VLRBs (Fig. 2b). Cloning the *VLRB* into a vector suitable for plant expression and *Agrobacterium*-mediated transformation of plants allows the targeting of specific proteins. In this study, we have successfully targeted the N-terminus of a bacterial effector from a plant pathogen, HopM1, by expressing an appropriate specific VLRB (VLR_M1_) *in planta* (Fig. 5d). VLR_M1_ interacted with HopM1 and not with an unrelated effector, and HopM1 failed to interact with a non-specific VLRB (VLR_TLR5_).

The different high-affinity sequences identified in this study for VLR_M1_ clones had very few polymorphisms. This lack of variability is not surprising, as only 3% nucleotide differences had been observed for 50 *VLRA* mRNAs (specific for hen egg lysozyme) [41], which are expressed by the lamprey T-like cells [25], but is in contrast with the finding that more than 30% of amino acids were different between seven VLRBs specific for the BclA *Bacillus anthracis* spore-coat protein [27]. The anti-BclA VLRB clones were screened as multivalent, secreted proteins that bound with high avidity, even though the monomeric subunits had low affinity. In contrast, the HopM1- and hen egg lysozyme-specific VLRs were isolated using yeast display to select for the highest affinity clones, which are uncommon in the repertoire and therefore, have more limited sequence diversity. The β-strands in the concave region of VLRB, which, except for the leucines of the LRR, are highly variable in sequence and confer binding specificity [16], were clearly divergent in amino acid sequence when comparing VLR_M1_, VLR_TLR5_ and VLR_HEL_. The overall amino acid sequence identity between VLR_M1_ and VLR_TLR5_ was 68%, and between VLR_M1_ and VLR_HEL_ was 77%, while the average amino acid identity between the variable amino acids (non-leucine) in the β-strands of LRR1, LRRRV, and LRRVe was only 20 and 19%, respectively.

VLRBs can be highly specific to the target being recognized, as VLRBs have been observed to differentiate between proteins that were 89% identical [27]. VLRBs have been shown to bind not only proteins but also carbohydrates [20, 40], and as such, VLRBs could be used to target specific carbohydrate moieties in plants. If desired, the binding specificity of the VLRs may be improved in vitro by random mutation of the amino acids responsible for the interaction in their corresponding LRRs. A more than a 1000-fold increase in binding has been observed using this method for identifying VLRs that recognize hen egg lysozyme [41]. In addition, VLRBs form high-avidity multimeric binding structures composed of 8–10 identical VLRBs as they are secreted [27]. The cysteines necessary for forming these higher order multimeric structures are not present in VLR_M1_, as this truncated protein lacks the invariant stalk region containing the cysteines and as such, is unable to form these multimeric structures. Multimeric secreted VLRBs with potentially higher affinity could be produced in plants by adding the stalk region to the plant-expressed VLRs.

Cytoplasmic expression of VLR_M1_ was relatively low, especially when compared to secreted VLR_M1_ (Fig. 3a, b). Expression of cytoplasmic immunoglobulin antibodies in plants has also encountered the same problem, as even when the immunoglobulin gene is highly transcribed, the accumulation of cytoplasmic immunoglobulin proteins is barely detectable in plants (with a more than 300-fold difference in protein concentration being observed when comparing cytoplasmic and secreted antibodies) [42]. Nanobodies^®^, the recombinant variable binding domain of heavy-chain only antibodies (V_H_H) from Camelids [43] offer another alternative to target plant proteins. However, high expression of nanobodies has only been observed in the apoplast [44] and chloroplasts [45]. The low antibody accumulation in the cytoplasm probably reflects the inability of the antibodies to form disulfide bonds in the reducing conditions of the cytoplasm [46], and would explain the low expression observed in this study for cytoplasmic VLR_M1_. Some proteins can still form disulfide bonds in the cytosol, especially under oxidative stress conditions [47], so it is still possible for a fraction of the VLR_M1_ proteins to fold properly in the cytoplasm.

In contrast to cytoplasmic VLR, VLR_M1_ targeted to the apoplast or the TGN expressed well (Fig. 3a, c). HopM1 has been observed to be localized to the TGN when the effector was expressed in transgenic plants [32]. In the future, plants with resistance against HopM1-expressing *P. syringae* strains could be developed by attaching SYP61 to VLR_M1_ and using VLR_M1_-ubiquitin ligase or NBS–VLR_M1_ fusions (see below). However, currently it is unknown if HopM1 localizes to the lumen of the TGN or to the surrounding cytoplasm. Based on this study, we predict that VLR-based binding with HopM1 or other TGN-targeted plant proteins would probably work only if these proteins are localized on the lumen side of the TGN, since disulfide bond formation is not as efficient in the cytoplasm.

Antibodies with immunoglobulin domains have been used in the past to target plant- or plant pathogen-expressed proteins. For example, immunoglobulins have been used to modulate *in planta* abscisic and gibberellic acid availability [48, 49]. Plant viruses have also been the target of antibodies, as targeting the coat protein, the RNA-dependent RNA polymerase, and the protease that cleaves the viral polyprotein precursor reduced *in planta* viral accumulation and symptom development [50–52]. Immunoglobulins against cell wall proteins of fungal pathogens have even been engineered to be linked to antifungal peptides, which ultimately lead to reduced symptom production in transgenic plants carrying the antibody fusion [53].

We anticipate several ways in which the LRR modules from VLRBs could be used for targeting proteins and/or modifying protein function in plants (Additional file 1: Figure S4). Firstly, since LRRs are used as modules for interaction in numerous plant proteins [14, 15], VLRBs could replace the binding domains of these proteins to generate chimeric VLRB-proteins with new binding specificities. The potential for using VLR technology is such that one can conceive creating plants with a pseudo-adaptive immune system, in which pattern-recognition receptors (PRR) and disease resistance proteins with new specificities against invading pathogens may be tailored as needed. VLRBs could replace the binding modules of receptor-like proteins and receptor-like kinases and the LRR domains of NBS–LRR proteins. Functional chimeric PRRs in which the LRR domains were swapped with those from a different PRR have already been characterized [54]. Chimeric proteins responding to new stimuli and causing developmental changes could also be created (e.g., VLR–BRI1 chimera, BRI1 is the brassinosteroid hormone receptor) [5]. So far, an unsuccessful attempt at constructing a functional PRR–VLR chimera, in which no plant responses against lysozyme (the antigen recognized by the VLRA) were observed, has been described [55]. The newly characterized structure of the PRR bound to its ligand [6] might help in the future in constructing a proper functional chimera.

Antigen-specific VLRBs could also be used to explore a phenotype of interest by inhibiting the activity of a protein by direct binding (as has been observed for enzyme inhibitors carrying LRR domains) [2, 13] or by targeting a protein for degradation and observing the change in the phenotype. Direct inhibition would require a VLRB with a much higher affinity than that of the enzyme for its substrate. Since VLRBs with binding affinities in the picomolar range have been observed [41], this would be theoretically possible. For targeting proteins for degradation, VLRBs may be incorporated into LRR-containing E3 ubiquitin ligases that target proteins for proteasomal degradation in the plant cell. Multiple possibilities exist for the future use of LRR-containing VLRs for targeting plant-expressed proteins.

## Conclusions

In this study, we have developed an original methodology for *in planta* targeting of proteins. This is achieved by immunizing lampreys with our target of interest, selecting VLRs with high-affinity for this protein target using flow cytometry and yeast surface display, and finally, expressing the target-specific VLR *in planta*. We found that VLR accumulation was higher when directed to the secretory pathway, although fusing the VLR to certain proteins, e.g., SYP61, might help stabilize them. With few systems available for *in planta* protein targeting, the VLR-based methodology offers the opportunity to bind and inhibit the function of specific plant proteins, and to construct chimeric proteins with new specificities in which the endogenous interacting domains are replaced by those of VLRs. This ultimately might facilitate the exploration and discovery of new phenotypes and mechanisms in plant biology.

## Methods

### Strains and antibiotics

*Agrobacterium tumefaciens* and *E. coli* strains (Additional file 2: Table S1) were grown on LB (Lennox) medium at 28–30 and 37 °C, respectively. Antibiotics were used at the following concentrations: 10 µg mL^−1^ gentamycin, 50 µg mL^−1^ kanamycin, 100 µg mL^−1^ rifampicin, and 50 µg mL^−1^ spectinomycin.

*Saccharomyces cerevisiae* strains (Additional file 2: Table S1) were grown on YPD (yeast extract peptone dextrose), SD-CAA (synthetic dextrose supplemented with casamino acids; 20 g L^−1^ dextrose, 6.7 g L^−1^ yeast nitrogen base, 100 mM sodium phosphate buffer, pH 6.0, and 5 g L^−1^ acid-hydrolyzed casamino acids lacking tryptophan) or SG-CAA (synthetic galactose supplemented with casamino acids; similar to SD-CAA but dextrose concentration is reduced to 1 g L^−1^ and 19 g L^−1^ galactose is included) media at 28–30 °C.

### Plant growth conditions

*Nicotiana benthamiana* plants were grown at 22–24 °C with a 12-h photoperiod. *Arabidopsis thaliana* plants were grown under a 12-h photoperiod, at 23 °C when the lights were on and at 21 °C when the lights were off.

### Sea lamprey culture

Sea lamprey larvae (*Petromyzon marinus*) of 12–15 cm in length were captured by commercial fishermen (Lamprey Services, Ludington, MI) and maintained in sand-lined, aerated aquariums at 16–20 °C. Lampreys were fed with brewer’s yeast. All lamprey experiments were approved by the Emory Institutional animal care and use committee (IACUC).

### Expression and purification of the N-terminus of HopM1

*Escherichia coli* BL21(DE3) pET28::*His*_*6*_-*hopM1*_*1*–*900*_ strain was grown at 37 °C until the O.D._600_ of a 200-mL culture reached 0.5. Protein expression was induced with the addition of 0.1 mM isopropyl β-d-1-thiogalactopyranoside (IPTG) and the culture was grown for 6 h at 22 °C. Cells were lysed by sonication (using the VirSonic 600 ultrasonic homogenizer from VirTis), centrifuged, and the supernatant was incubated with Ni–NTA agarose resin (QIAGEN) to capture polyhistidine-tagged proteins. Proteins were eluted from the resin with 0.5 M imidazole, and the sample diluted with 3 volumes of 30 mM Tris–HCl pH 8.3. A second-step purification of HopM1_1–300_ used the UNO S-1 ion exchange chromatographic column (Bio-Rad) and the BioLogic DuoFlow™ chromatography system (Bio-Rad). HopM1_1–300_ was eluted from the ion exchange column with 433 mM NaCl, and then desalted and resuspended in phosphate-buffered saline (PBS), pH 7.6, by dialysis.

### Lamprey immunization

Lampreys respond to particulate antigens, such as intact viruses, bacteria and mammalian cells, but soluble proteins are weakly immunogenic on their own. Several adjuvants have been developed for vertebrates to enhance the immune response, most of which are ineffective in lampreys. Although complete Freund’s adjuvant can enhance the VLRB response, in our hands, it is toxic to lamprey larvae resulting in a high mortality rate. Given that mammalian cells are immunogenic, we determined that protein antigens or haptenated proteins covalently coupled to human Jurkat T cells by amine linkage reproducibly induced VLRB responses to both protein and hapten epitopes without toxicity. Accordingly, HopM1_1–300_ was conjugated to Jurkat T cells before lamprey immunization.

For HopM1_1–300_ conjugation, 10^8^ Jurkat T cells were fixed overnight in 4% paraformaldehyde. The fixed Jurkat T cells were washed in 20 mM MES, pH 5.5, and then activated for amine conjugation with EDC/NHS (1-ethyl-3-(-3-dimethylaminopropyl) carbodiimide hydrochloride/*N*-hydroxysuccinimide) for 20 min at room temperature. Cells were briefly washed in PBS, and then 0.2 mg of HopM1_1–300_ was added to the pelleted EDC/NHS-activated cells for 3 h at room temperature. After conjugation, HopM1_1–300_-conjugated cells were washed once with PBS containing 10 mM Tris–HCl, pH 7.5; and stored at 4 °C until needed for lamprey immunization.

Sea lamprey larvae were sedated with 0.1 g L^−1^ of tricainemethanesulfonate (Tricaine-S; Western Chemical, Inc.) before injection into the coelomic cavity with 20 µg of recombinant HopM1_1–300_ covalently conjugated to formaldehyde-fixed Jurkat T cells. Three lampreys were immunized for a total of 3 times at 2-week intervals. Two weeks after the final immunization, the lampreys were euthanized with 1 g L^−1^ of tricainemethanesulfonate and exsanguinated by tail severing. Blood was collected in a 30 mM EDTA solution (serving as an anticoagulant), and plasma and leukocytes were separated using a 55% Percoll gradient. The plasma samples were used to measure the lamprey VLRB response to immunization by ELISA, while the leukocytes were stored in RNA*later* ^®^ (Thermo Fisher Scientific) at −80 °C until needed for *VLRB* cDNA library cloning.

### Enzyme-linked immunosorbent assay (ELISA)

ELISA plates coated with 5 µg mL^−1^ of recombinant HopM1_1–300_ were blocked with 2% skim milk in TBST (20 mM Tris–HCl, 150 mM NaCl, and 0.1% Tween-20, pH 7.5), before incubation with serial dilutions of plasma from HopM1-immunized lampreys or control non-immunized plasma. VLRB binding was detected with an α-VLRB mouse monoclonal antibody (4C4) [23] and an alkaline phosphatase (AP)-conjugated goat α-mouse IgG polyclonal antibody (SouthernBiotech; this secondary antibody binds to the α-VLRB antibody). In between each incubation period, five washes with TBST were performed. Enzyme activity was detected after addition of an AP substrate (*p*-nitrophenyl phosphate, SIGMA-Aldrich), after which plates were incubated for 30 min at room temperature, followed by AP enzyme inactivation with 0.1 M NaOH. Absorbance readings at 405 nm were collected and the data was graphed using GraphPad PRISM software.

### VLRB library construction

RNA was isolated from total leukocytes samples collected from lampreys immunized with HopM1_1–300_ using the RNeasy kit (QIAGEN). RNA was reverse transcribed into cDNA using SuperScript^®^ III reverse transcriptase (Invitrogen™) and oligo-dT primers. *VLRB* transcripts were amplified from the leukocyte cDNA by nested PCR using high-fidelity DNA polymerase (Novagen^®^). The first round of PCR used primers that annealed to the 5′ and 3′ untranslated regions of *VLRB*, AVL001 and AVL002 (Additional file 2: Table S2), respectively. The second round of PCR used primers that amplified only the *VLRB* antigen-binding domain, from the N-terminal LRR to the C-terminal LRR (primers AVL003 and AVL004, respectively). These primers had approximately 50 bp of sequence homology to the yeast surface display (YSD) vector (pCT-ESO) for cloning by in vivo homologous recombination in transfected yeast cells.

The pCT-ESO plasmid adds a c-Myc epitope at the end of the *VLRB* insert and anchors the VLRB to the yeast cell wall by fusing the protein to Aga2p. *VLRB* expression in this system is controlled under a galactose-inducible promoter. To clone the *VLRB* cDNAs, the *BDNF* gene from the pCT-ESO-BDNF plasmid [56] was removed by restriction digestion with NheI and BamHI, and NcoI digestion (New England BioLabs^®^ Inc.) to eliminate the *BDNF* insert.

For VLR library transformation, tryptophan-auxotroph *S. cerevisiae* strain EBY100 was grown to the log phase in YPD media at 30 °C until the O.D._600_ reached 1.0. Cells were washed with H_2_O, and incubated in 10 mM Tris–HCl, 10 mM DTT, 100 mM lithium acetate, pH 7.6, at 225 rpm and 30 °C for 20 min. After incubation, yeast cells were washed with H_2_O and resuspended in 1 M sorbitol to a concentration of 10^9^ cell mL^−1^. Two hundred µL of yeast cells, 1 µg of digested pCT-ESO vector and 2 µg of the purified VLRB PCR product were mixed and electroporated at 2.5 kV using a Micropulser™ electroporator (Bio-Rad). The total number of transformants was estimated to be 1.1 × 10^6^ VLRB clones. Aliquots of the transformed yeast library were stored at −80 °C in 15% glycerol.

### Yeast surface display

Two rounds of enrichment for HopM1_1–300_-binding VLRBs using Fluorescence-activated and Magnetic-activated cell sorting (FACS and MACS, respectively) were performed. For HopM1_1–300_ biotinylation, the EZ-link NHS-LC-LC-biotin kit (Thermo Fisher Scientific) was used.

For determination of binding to HopM1 of individual yeast colonies, an overnight yeast culture was diluted into fresh SD-CAA medium and grown at 30 °C for 3 h. Culture was centrifuged and resuspended in SG-CAA medium (to induce VLRB expression) and further grown for 48 h at 20 °C. Fifty µL of yeast cells were washed once in staining buffer (PBS pH 7.4 with 1% BSA) and then incubated for 30 min with 50–250 nM biotinylated HopM1_1–300_. Cells were washed thrice with staining buffer, and then incubated for 30 min at 4 °C in staining buffer with 5 µg mL^−1^ mouse α-Myc-Alexa Fluor^®^ 488 (clone 4A6; EMD Millipore) and 2.7 µg mL^−1^ streptavidin, R-phycoerythrin conjugate (Invitrogen™). HopM1_1–300_ binding was evaluated using the BD Accuri™ C6 flow cytometer (BD BioSciences) and a 488 nM excitation laser. For detection of Alexa Fluor^®^ 488 fluorescence, a 533/30 nm filter (FL-1 channel) was used. For detection of phycoerythrin, a 585/40 nm filter (FL-2 channel) was used. The FL-1 channel was corrected by subtracting 5% of the FL-2 channel, while the FL-2 channel was corrected by subtracting 6.2% of the FL-1 channel.

To determine which events captured by the flow cytometer corresponded to VLRB binding of HopM1, the fluorescence intensity in the Alexa Fluor^®^ 488 versus phycoerythrin plot was divided into four quadrants. The quadrant in the left lowermost corner represents those events in which neither *VLRB* expression nor HopM1 binding occurred, and its limits were determined by using samples in which *VLRB* expression was not induced. The quadrant in the right uppermost corner represents those events in which both VLRB expression and HopM1 binding occurred.

### Cloning

VLRBs were amplified from individual colonies of yeast surface display libraries using KOD hot start DNA polymerase (Novagen^®^) and Zymolase (Zymo Research), primers AVL005 and AVL006 (Additional file 2: Table S2), and a 30 min incubation at 37 °C prior to PCR (for the Zymolase to degrade the yeast cell wall). Purified PCR products were used as DNA templates with Phusion^®^ high-fidelity DNA polymerase (Thermo Fisher Scientific) and primers AVL007 and AVL008 to clone into the pCR™8/GW/TOPO^®^ entry vector (Invitrogen™). Prior to cloning, addition of adenine overhangs was performed using GoTaq^®^ DNA polymerase (Promega).

The nucleotide sequence corresponding to the N-terminus of HopM1 (*PSPTO_1375*; amino acids 1–300) was amplified from *P. syringae* pv. *tomato* (*Pst*) DC3000 genomic DNA using Phusion^®^ high-fidelity DNA polymerase (Thermo Fisher Scientific) and primers AVL009 and AVL010. The signal peptide sequence of *AtPR1* (*At2g14610*; *SP*) was amplified from *A. thaliana* cDNA with primers AVL011 and AVL012. *SYP61* (*At1g28490*) was amplified from *A. thaliana* cDNA with primers AVL013 and AVL014. *mRFP1* was amplified from plasmid pGWB554 [57] with primers AVL015 and AVL016. All these DNA sequences were cloned into pCR8™/GW/TOPO^®^. *Pst* DC3000 *hopK1* (*PSPTO_0044*) was amplified with primers AVL017 and AVL018 from *Pst* DC3000 genomic DNA and cloned into pDONR207. The nucleotide sequence corresponding to the N-terminus of HopM1 was also amplified using primers AVL019 and AVL020, cloned into plasmid pGEM^®^-T-Easy (Promega Corporation) and then cloned into pET28a by using the restriction enzymes NdeI and EcoRI (New England BioLabs^®^ Inc.).

To create a fusion between the signal peptide of AtPR1 (SP) and VLR_M1_, *SP* was amplified using primers AVL021 and AVL022 such that the amplicon had overhangs that were identical in sequence to the pCR8 vector on the 5′ end and VLR_M1_ on the 3′ end. The PCR product was purified and used in a second round of PCR with pCR8::*VLR*_*M1*_; both templates had overlapping sequences, so that after the second PCR a single plasmid containing the signal peptide fused to the VLR would be produced. After amplification, removal of the original template plasmid was performed using restriction enzyme DpnI (New England BioLabs^®^ Inc.).

Overlap-extension PCR (OE-PCR) was used to create a fusion between *SP* and *hopM1* using primers AVL011 and AVL023 to amplify *SP* with overlaps, and AVL024 and AVL010 to amplify *hopM1* with overlaps. The purified PCR products were used on a second PCR with primers AVL011 and AVL010 to create *SP*-*hopM1*_*1*–*900*_, which was then cloned into pCR8™/GW/TOPO^®^. OE-PCR was also used to create fusions between *SP* and *VLR*_*TLR5*_ (using primers AVL011 and AVL022, and AVL025 and AVL008), and *SYP61* and *VLR*_*M1*_ (using primers AVL007 and AVL026, and AVL027 and AVL014).

*VLR*_*M1*_, *VLR*_*TLR5*_, *mRFP1*, *A. thaliana SYP61* and *SP*, *Pst* DC3000 *hopM1* and *hopK1*, and fusion proteins were cloned into destination vectors pGWB514, pGWB517, and pGWB554 [57]; and pDest-35S-X-YFP-6xHis [58] using Gateway^®^ recombination technology (Invitrogen™).

### Alignment and 3-D structure modeling

Amino acid alignment was performed using MegAlign™ (DNASTAR^®^), and the alignment was graphed using BoxShade (Hofmann and Baron). Protein domains were predicted using the SMART tool [59].

3-D structure modeling was performed using SWISS-MODEL (Swiss Institute of Bioinformatics) and the structure of a VLRB specific for α-hen egg white lysozyme (VLR_HEL_; 3g3a) [16].

### Transient *in planta* expression of VLRBs in *Nicotiana benthamiana*

*Agrobacterium tumefaciens* strains were grown overnight in LB with appropriate antibiotics, washed twice with 10 mM MgCl_2_, 10 mM MES (pH 5.6); and resuspended in the same buffer containing 200 µM acetosyringone to an O.D._600_ of 0.2, except for the YFP culture, whose O.D._600_ was adjusted between 0.010 and 0.025. Cultures were incubated in the dark for 3 h at room temperature, after which 5- to 6-week old *N. benthamiana* plants were infiltrated using a needleless syringe. Forty-eight hours post-infiltration, samples were collected for protein extraction or visualization on the microscope.

### Stable expression of VLRBs in *Arabidopsis thaliana*

To generate transgenic *A. thaliana* plants, the floral dip method [60] was used. After seeds were collected, transformants were selected in ½ concentration Linsmaier and Skoog (LS) medium with 25 µg mL^−1^ hygromycin. Genomic DNA from putative transformants was extracted using the method of Edwards et al. [61] and the presence of the transgene confirmed by PCR using primers AVL007 and AVL014.

### Protein extraction

Frozen leaf tissue was ground using 3-mm zirconium oxide beads (Glen Mills Inc.) and the TissueLyser II homogenizer (QIAGEN) or, for larger quantities, using a mortar and a pestle. Ground tissue was incubated with 3 volumes (µL) of extraction buffer (0.5–1.0% Triton X-100, 150 mM NaCl, 100 mM Tris–HCl pH 7.5, 10 mM dithiothreitol [DTT], 5 mM EDTA, and protease inhibitor cocktail for plant cell and tissue extracts from SIGMA-Aldrich) per mg of tissue for 10 min at 4 °C, after which the sample was centrifuged to remove the tissue debris. Protein concentration was determined using the Bradford method (Bio-Rad protein assay), so that every sample within an experiment was adjusted to have the same concentration.

### Electrophoresis and Western blotting

Polyacrylamide gel electrophoresis was performed using the NuPAGE^®^ electrophoresis system (Thermo Fisher Scientific) and NuPAGE^®^ Novex^®^ 4–12% Bis–Tris gels following manufacturer’s recommendations (45 min at 200 V and 120 mA [maximum]). Protein transfer was confirmed by staining the PVDF membrane with Ponceau S stain (0.1% Ponceau S in 5% acetic acid). Western blotting was performed with the following antibodies: α-c-Myc and α-GFP (abcam^®^), α-HA-HRP (3F10; Roche), and α-rabbit IgG-HRP (Thermo Fisher Scientific). For chemiluminescent detection, the SuperSignal™ West Dura extended duration substrate (Thermo Fisher Scientific) and Blue Ultra Autoradiography film (GeneMate) were used.

Staining of gels during HopM1_1–300_ purification was performed with Denville Blue™ protein stain (Denville Scientific Inc.) following manufacturer’s recommendations.

### Co-immunoprecipitation

Proteins were extracted by incubating ground tissue in extraction buffer (0.5% Triton X-100, 150 mM NaCl, 50 mM Tris–HCl pH 7.5, 0.5 mM EDTA, and protease inhibitor cocktail for plant cell and tissue extracts from SIGMA-Aldrich) for 1 h at 4 °C. No reducing agent (e.g., DTT) was included in the extraction buffer. Total protein immunoprecipitated was adjusted to be the same for every sample within an experiment. Extracted proteins (diluted to have a Triton X-100 concentration of 0.2%) were incubated with 20 µL of GFP-nAb™ (Allele Biotechnology), α-c-Myc (SIGMA-Aldrich), or α-HA (clone HA-7, SIGMA-Aldrich) agarose beads for 1 h at 4 °C. Beads were centrifuged and washed 4 times, after which immunoprecipitated proteins were released from the beads by resuspending them in 75 µL of LDS buffer (Thermo Fisher Scientific) and incubating for 10 min at 70 °C.

### Confocal and epifluorescent microscopy

Confocal images were taken with the Olympus FluoView™ FV1000 confocal microscope. For YFP detection, the excitation used a 515 nm argon gas laser (10 mW, at 10% intensity), while the emission was collected between 530 and 569 nm. For mRFP1 detection, the excitation used the 559 nm solid-state diode laser (10 mW, at 10% intensity), coupled with an emission collected between 570 and 600 nm. Images were visualized with a 40×-magnification oil-immersion objective that had a numerical aperture (NA) of 1.3. Images were acquired at a voltage (HV) lower than one that gave fluorescence signal for an untransformed control and at which very few pixels for the image were starting to saturate. In all images, the offset parameter was adjusted to 10% and the line Kalman integration to 3.

Epifluorescent images were acquired with the Olympus IX71 inverted microscope equipped with a 120-W metal halide lamp and a YFP filter (Semrock). The filter had an excitation of 500/24 nm and an emission of 542/27 nm. Images were visualized with a 10×-magnification objective and were acquired at an exposure time at which the untransformed control did not show any autofluorescence.

## Additional files

**Additional file 1: Figure S1.** Production of VLRB antibodies after HopM1_1–300_ immunization in lampreys. ELISA results for VLRB production from dilutions of plasma from three lampreys immunized with HopM1_1–300_ conjugated to Jurkat T cells and a control non-immunized lamprey (naïve). Binding of VLRBs to HopM1_1–300_-coated plates was detected with a mouse monoclonal antibody and an alkaline peroxidase-conjugated goat α-mouse IgG polyclonal antibody. Absorbance at 405 nm (A_405_) was measured 30 min after addition of an alkaline peroxidase substrate. Lamprey-1 showed the highest response to HopM1_1–300_. **Figure S2**. VLRBs can be targeted to intracellular compartments. Visualization of intracellular accumulation of YFP, syntaxin SYP61 (*At1g28490*), and VLR_M1_ fused to SYP61 in *N. benthamiana*. Images were taken with the Olympus IX71 inverted microscope using the YFP filter (excitation 500/24, emission 542/27). White bar length represents 50 µm. Image brightness increased 15% for YFP, and 20% for the other 2 images. Notice how the YFP fluorescence pattern is similar for SYP61 (which localizes to the early endosome/*trans*-Golgi network) [34, 37] and for VLR_M1_-SYP61. **Figure S3.** *In planta* interaction of HopM1 with VLR_M1_. Co-immunoprecipitation (co-IP) of HopM1 and its corresponding VLR in *Nicotiana benthamiana*. Interactions between HopM1 and VLR_M1_ were tested with both proteins fused to 2 different epitope tags (HA and c-Myc). Highlighted in orange are those proteins detected in the Western blot, while in black are those proteins also expressed but not detected. As negative controls for the co-immunoprecipitations, different proteins that had low or no expression were co-expressed with HopM1 or VLR_M1_ (data not shown). No reducing agents were used in the buffers. Abbreviations used: VLR_M1_ = SP-VLR_M1_, and M_1–300_ = SP-HopM1_1–300_. **a** Total protein input of HA and c-Myc tagged proteins. Proteins were detected with α-HA and α-c-Myc antibodies, respectively. Ponceau S staining of the PVDF membrane is shown below the Western blot image. **b** Immunoprecipitation (IP) using α-HA agarose beads. The IP (α-HA antibodies) and co-IP (α-c-Myc antibodies) Western blots are shown. **c** Reciprocal immunoprecipitation using α-c-Myc agarose beads. The IP (α-c-Myc antibodies) and co-IP (α-HA antibodies) Western blots are shown. **Figure S4.** Hypothetical modifications to VLRs to diversify their *in planta* use. Abbreviations used: NBS = nucleotide-binding site, RLK = receptor-like kinase, RLP = receptor-like protein, and VLR = variable lymphocyte receptor.

**Additional file 2: Table S1.** Strains used in this study. **Table S2.** Primers used in this study.
