# Supplementary material for: Leucine-rich-repeat-containing variable lymphocyte receptors as modules to target plant-expressed proteins
Source: Plant Methods. 2017 Apr 19;13:29. doi: 10.1186/s13007-017-0180-8 (PMC5395774; doi:10.1186/s13007-017-0180-8)
Supplement: Supplementary file 1 — Additional file 1: Figure S1. Production of VLRB antibodies after HopM11–300 immunization in lampreys. ELISA results for VLRB production from dilutions of plasma from three lampreys immunized with HopM11–300 conjugated to Jurkat T cells and a control non-immunized lamprey (naïve). Binding of VLRBs to HopM11–300-coated plates was detected with a mouse monoclonal antibody and an alkaline peroxidase-conjugated goat α-mouse IgG polyclonal antibody. Absorbance at 405 nm (A405) was measured 30 min after addition of an alkaline peroxidase substrate. Lamprey-1 showed the highest response to HopM11–300. Figure S2. VLRBs can be targeted to intracellular compartments. Visualization of intracellular accumulation of YFP, syntaxin SYP61 (At1g28490), and VLRM1 fused to SYP61 in N. benthamiana. Images were taken with the Olympus IX71 inverted microscope using the YFP filter (excitation 500/24, emission 542/27). White bar length represents 50 µm. Image brightness increased 15% for YFP, and 20% for the other 2 images. Notice how the YFP fluorescence pattern is similar for SYP61 (which localizes to the early endosome/trans-Golgi network) [34, 37] and for VLRM1-SYP61. Figure S3. In planta interaction of HopM1 with VLRM1. Co-immunoprecipitation (co-IP) of HopM1 and its corresponding VLR in Nicotiana benthamiana. Interactions between HopM1 and VLRM1 were tested with both proteins fused to 2 different epitope tags (HA and c-Myc). Highlighted in orange are those proteins detected in the Western blot, while in black are those proteins also expressed but not detected. As negative controls for the co-immunoprecipitations, different proteins that had low or no expression were co-expressed with HopM1 or VLRM1 (data not shown). No reducing agents were used in the buffers. Abbreviations used: VLRM1 = SP-VLRM1, and M1–300 = SP-HopM11–300. a Total protein input of HA and c-Myc tagged proteins. Proteins were detected with α-HA and α-c-Myc antibodies, respectively. Ponceau S staining of the PVDF memb [file 13007_2017_180_MOESM1_ESM.pdf]

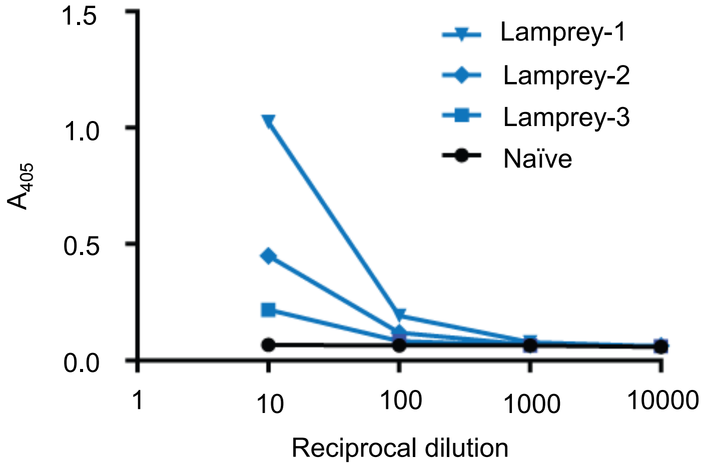

Additional file 1:

Figure S1. Production of VLRB antibodies in lampreys after HopM$1_{1\text{-}300}$ immunization.

ELISA results for VLRB production from dilutions of plasma from three lampreys immunized with HopM$1_{1\text{-}300}$ conjugated to Jurkat T cells and a control non-immunized lamprey (naïve). Binding of VLRBs to HopM$1_{1\text{-}300}$-coated plates was detected with a mouse monoclonal antibody and an alkaline peroxidase conjugated goat anti-mouse IgG polyclonal antibody. Absorbance at 405 nm (A_{405}) was measured 30 minutes after addition of an alkaline peroxidase substrate. Lamprey-1 showed the highest response to HopM$1_{1\text{-}300}$.

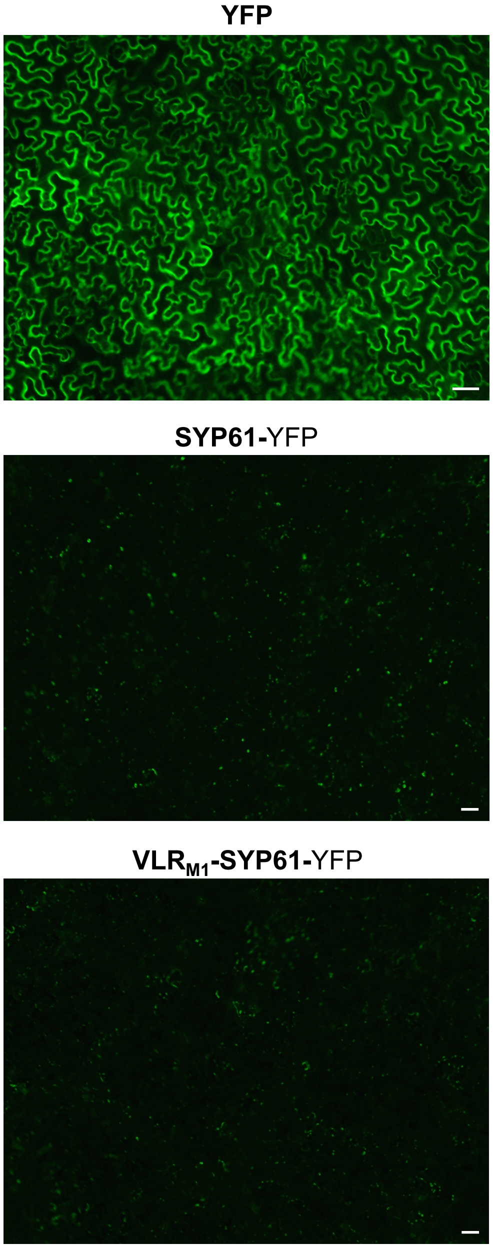

Figure S2. VLRBs can be targeted to intracellular compartments.

Visualization of intracellular accumulation of YFP, syntaxin SYP61 (*At1g28490*), and VLR$_{M1}$ fused to SYP61 in *Nicotiana benthamiana*. Images were taken with the Olympus IX71 inverted microscope using the YFP filter (excitation 500/24, emission 542/27). White bar length represents 50 µm. Image brightness increased 15% for YFP, and 20% for the other 2 images. Notice how the YFP fluorescence pattern is similar for SYP61 (which localizes to the early endosome/trans-Golgi network; Sanderfoot *et al.* 2001, Stefano *et al.* 2010) and for VLR$_{M1}$-SYP61.

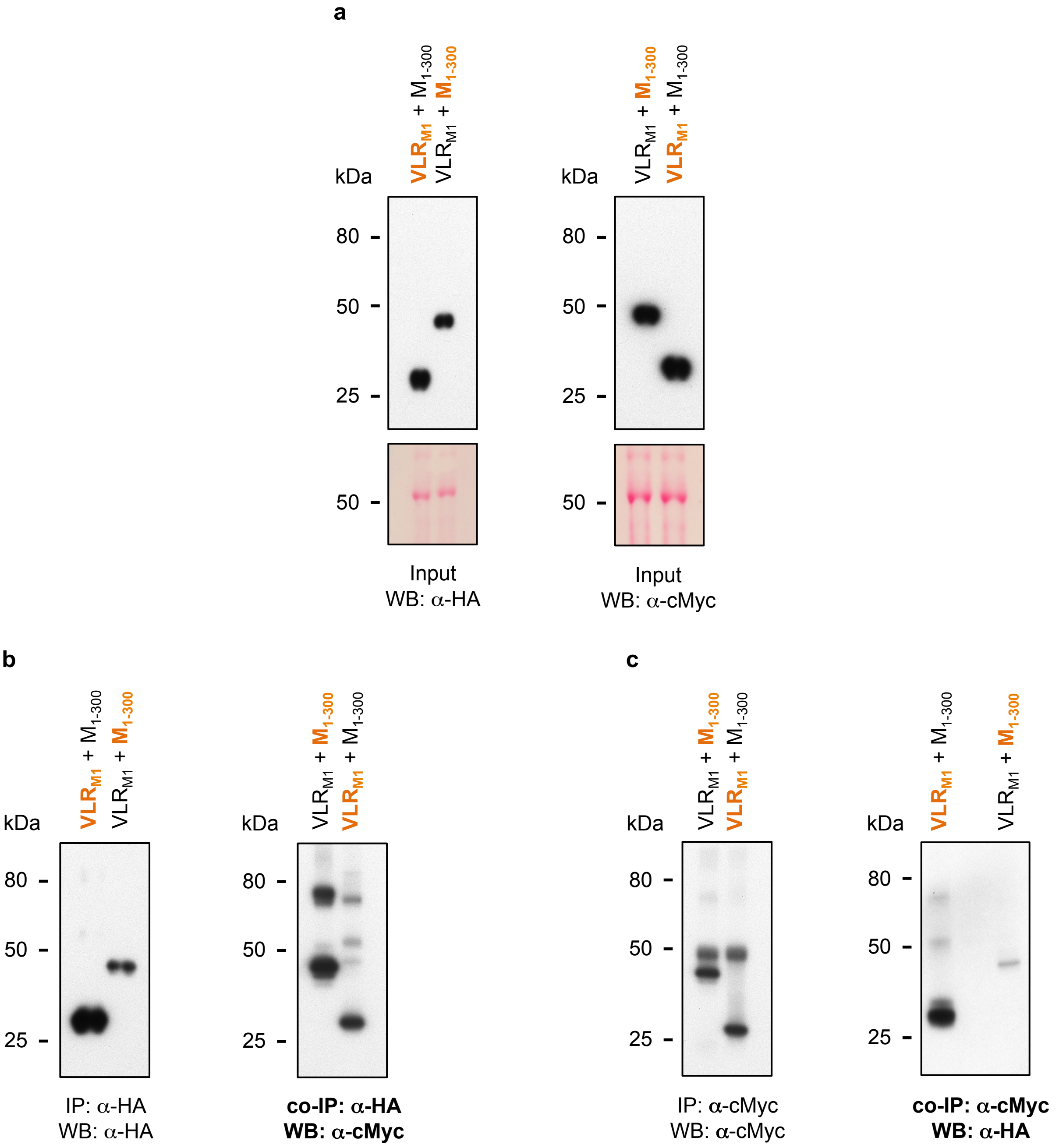

Figure S3. *In planta* interaction of HopM1 with VLR$_{M1}$.

Co-immunoprecipitation (co-IP) of HopM1 and its corresponding VLRB in *Nicotiana benthamiana*. Interactions between HopM1 and VLR$_{M1}$ were tested with both proteins fused to 2 different epitope tags (HA and cMyc). Highlighted in orange are those proteins detected in the Western blot, while in black are those proteins also expressed but not detected. As negative controls for the co-immunoprecipitations, different proteins that had low or no expression were co-expressed with HopM1 or VLR$_{M1}$ (data not shown). No reducing agents were used in the buffers. Abbreviations used: VLR$_{M1}$ = SP-VLR$_{M1}$, and M$_{1\text{-}300}$ = SP-HopM1$_{1\text{-}300}$.

a Total protein input of HA and c-Myc tagged proteins. Proteins were detected with α-HA and α-cMyc antibodies, respectively. Ponceau S staining of the PVDF membrane is shown below the Western blot image.

b Immunoprecipitation (IP) using α-HA agarose beads. The IP (α-HA antibodies) and co-IP (α-cMyc antibodies) Western blots are shown.

c Reciprocal immunoprecipitation using α-cMyc agarose beads. The IP (α-cMyc antibodies) and co-IP (α-HA antibodies) Western blots are shown.

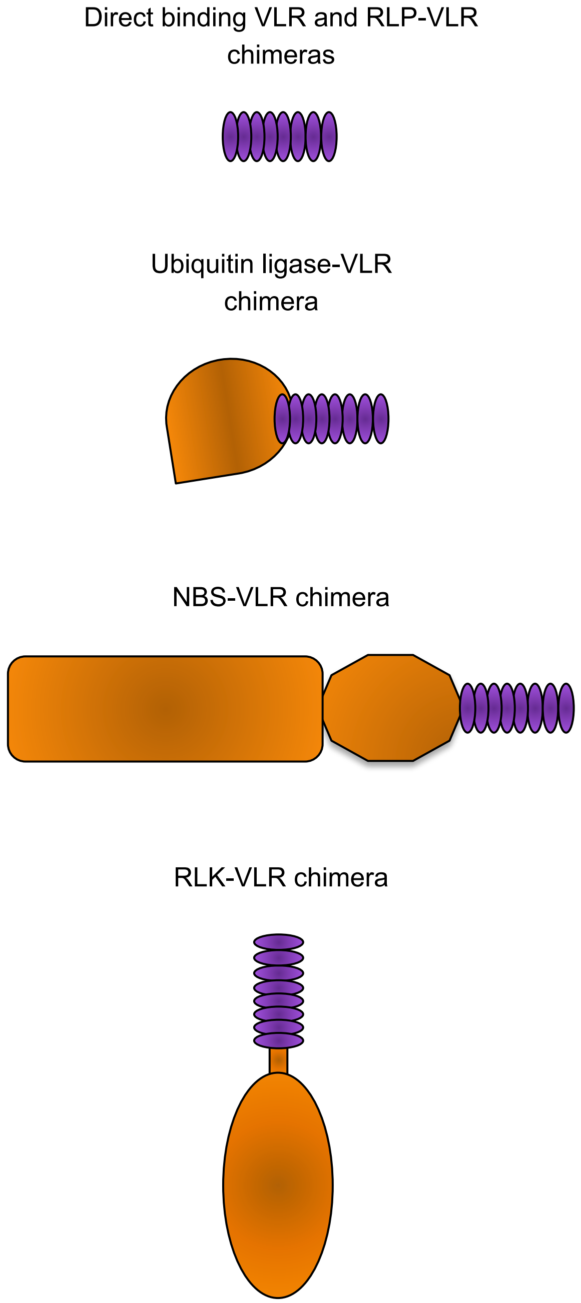

Figure S4. Hypothetical modifications to VLRs to diversify their *in planta* use.

Abbreviations used: NBS = nucleotide-binding site, RLK = receptor-like kinase, RLP = receptor-like protein, and VLR = variable lymphocyte receptor.
