# Supplementary material for: Leucine-rich-repeat-containing variable lymphocyte receptors as modules to target plant-expressed proteins
Source: Plant Methods. 2017 Apr 19;13:29. doi: 10.1186/s13007-017-0180-8 (PMC5395774; doi:10.1186/s13007-017-0180-8)
Supplement: Supplementary file 2 — Additional file 2: Table S1. Strains used in this study. Table S2. Primers used in this study. [file 13007_2017_180_MOESM2_ESM.pdf]

ADDITIONAL FILE 2:

Table S1. Strains used in this study.

Name	Comments
A. tumefaciens GV3101	
A. tumefaciens GV3101 pDest-35S-X-YFP-6xHis::*hopK1-YFP-His*$_6$	HopK1 with a YFP and a hexahistidine tag at the C-terminus of the protein.
A. tumefaciens GV3101 pDest-35S-X-YFP-6xHis::*SP*$_{PR1}$-*hopM1*$_{1\text{-}900}$-*YFP-His*$_6$	SP$_{PR1}$ (signal peptide of AtPR1) fused to the N-terminus of HopM1 (amino acids 1 to 300). Protein carries a YFP and a hexahistidine tag at the C-terminus of the protein.
A. tumefaciens GV3101 pDest-35S-X-YFP-6xHis::*SP*$_{PR1}$-*VLR*$_{M1}$-*YFP-His*$_6$	SP$_{PR1}$ (signal peptide of AtPR1) fused to VLR$_{M1}$. Protein carries a YFP and a hexahistidine tag at the C-terminus of the protein.
A. tumefaciens GV3101 pDest-35S-X-YFP-6xHis::*SP*$_{PR1}$-*VLR*$_{TLR5}$-*YFP-His*$_6$	SP$_{PR1}$ (signal peptide of AtPR1) fused to VLR$_{TLR5}$. Protein carries a YFP and a hexahistidine tag at the C-terminus of the protein.
A. tumefaciens GV3101 pDest-35S-X-YFP-6xHis::*SYP61-YFP-His*$_6$	SYP61 with a YFP and a hexahistidine tag at the C-terminus of the protein.

Name	Comments
A. tumefaciens GV3101 pDest-35S-X-YFP-6xHis::*VLR_{M1}-SYP61-YFP-His_6*	VLR_{M1} fused to a flexible linker (Gly-Ser-Ala-Gly-Ser-Ala-Ala-Gly-Ser-Gly-Glu-Phe), and fused to SYP61. Protein carries a YFP and a hexahistidine tag at the C-terminus of the protein.
A. tumefaciens GV3101 pDest-35S-X-YFP-6xHis::*VLR_{M1}-YFP-His_6*	VLR_{M1} with a YFP and a hexahistidine tag at the C-terminus of the protein.
A. tumefaciens GV3101 pGWB514::*SP_{PR1}-hopM$1_{1\text{-}900}$-HA_3*	SP_{PR1} (signal peptide of AtPR1) fused to the N-terminus of HopM1 (amino acids 1 to 300). Protein carries three HA tags at the C-terminus of the protein.
A. tumefaciens GV3101 pGWB514::*SP_{PR1}-VLR_{M1}-HA_3*	SP_{PR1} (signal peptide of AtPR1) fused to VLR_{M1}. Protein carries three HA tags at the C-terminus of the protein.
A. tumefaciens GV3101 pGWB514::*SYP61-HA_3*	SYP61 with three HA tags at the C-terminus of the protein.
A. tumefaciens GV3101 pGWB514::*VLR_{M1}-HA_3*	VLR_{M1} with three HA tags at the C-terminus of the protein.
A. tumefaciens GV3101 pGWB514::*VLR_{M1}-SYP61-HA_3*	VLR_{M1} fused to a flexible linker (Gly-Ser-Ala-Gly-Ser-Ala-Ala-Gly-Ser-Gly-Glu-Phe), and fused to SYP61. Protein carries three HA tags at the C-terminus of the protein.
A. tumefaciens GV3101 pGWB517::*hopK1-$cMyc_4$*	HopK1 with four cMyc tags at the C-terminus of the protein.

Name	Comments
A. tumefaciens GV3101 pGWB517::*mRFP1-cMyc*$_4$	mRFP1 with four cMyc tags at the C-terminus of the protein.
A. tumefaciens GV3101 pGWB517::*SP*$_{PR1}$*-hopM1*$_{1\text{-}900}$*-cMyc*$_4$	SP$_{PR1}$ (signal peptide of AtPR1) fused to the N-terminus of HopM1 (amino acids 1 to 300). Protein carries four cMyc tags at the C-terminus of the protein.
A. tumefaciens GV3101 pGWB517::*SP*$_{PR1}$*-VLR*$_{M1}$*-cMyc*$_4$	SP$_{PR1}$ (signal peptide of AtPR1) fused to VLR$_{M1}$. Protein carries four cMyc tags at the C-terminus of the protein.
A. tumefaciens GV3101 pGWB517::*SP*$_{PR1}$*-VLR*$_{TLR5}$*-cMyc*$_4$	SP$_{PR1}$ (signal peptide of AtPR1) fused to VLR$_{TLR5}$. Protein carries four cMyc tags at the C-terminus of the protein.
A. tumefaciens GV3101 pGWB554::*SP*$_{PR1}$*-mRFP1*	SP$_{PR1}$ (signal peptide of AtPR1) with an mRFP1 at the C-terminus of the protein.
A. tumefaciens GV3101 pGWB554::*SP*$_{PR1}$*-VLR*$_{M1}$*-mRFP1*	SP$_{PR1}$ (signal peptide of AtPR1) fused to VLR$_{M1}$. Protein carries an mRFP1 at the C-terminus of the protein.
A. tumefaciens pEarleyGate104	Gateway cassette removed by XmaI restriction. YFP protein expressed under a CaMV 35S promoter.
E. coli BL21(DE3) pET28a::*His*$_6$*-HopM1*$_{1\text{-}900}$	N-terminus of HopM1 (amino acids 1 to 300) with a hexahistidine at the N-terminus of the protein.
E. coli DH5α	
E. coli TOP10	

Name	Comments
S. cerevisiae EBY100 pCT-ESO::*VLR_{M1}* library	Yeast surface display library selected for VLRs binding to the N-terminus of HopM1 (amino acids 1 to 300).
S. cerevisiae EBY100 pCT-ESO::*VLR_{TLR5}* library	Yeast surface display library selected for VLRs binding to TLR5.

Table S2. Primers used in this study.

Name	Sequence 5' ⟶ 3'	Comments
AVL001	CTCCGCTACTCGGCCTGCA	Aligns to the 5' UTR of *VLRB*.
AVL002	CCGCCATCCCCGACCTTTG	Aligns to the 3' UTR of *VLRB* In the reverse orientation.
AVL003	**GGTGGAGGAGGCTCTGGTGGAGGCGGTAGCGGAGGCGGAGGGTCGGCTAGC**GCATGTCCCTCGCAGTG	Aligns to the N-terminal LRR (LRRNT) of *VLRB*. In bold is 51 bp of sequence identity to the YSD vector that was added to the primer.
AVL004	**GATCTCGAGCTATTACAAGTCCTCTTCAGAAATAAGCTTTTGTTCGGATCCC**GTGGTCGTAGCAACGTAG	Aligns to the C-terminal LRR (LRRCT) of *VLRB* in the reverse orientation. In bold is 52 bp of sequence identity to the YSD vector that was added to the primer.
AVL005	ACGACGTTCCAGACTACG	Aligns to pCT-ESO plasmid.
AVL006	TACAGTGGGAACAAAGTCG	Aligns to pCT-ESO plasmid.
AVL007	ATGGCATGTCCCTCGCAGTGTTC	Aligns to *VLRB* after the signal peptide. Primer adds an ATG start codon at the 5' end.
AVL008	CGTGGTCGTAGCAACGTAGC	Aligns to the 3' end of *VLRB* in the reverse orientation without including the stalk region.

Name	Sequence 5' ⟶ 3'	Comments
AVL009	ATGATCAGTTCGCGGATCGG	Aligns to nucleotides 1 to 20 of *hopM1* from *Pst* DC3000.
AVL010	TGCACCTTTCCAGCCACCCA	Aligns to nucleotides 881 to 900 of *hopM1* from *Pst* DC3000 in the reverse orientation.
AVL011	ATGAATTTTACTGGCTATTCTC	Aligns to nucleotides 1 to 22 bp of *AtPR1* (At2g14610).
AVL012	ATCTTGAGCTTTCGAGGGAA	Aligns to nucleotides 65 to 84 bp of *AtPR1* (At2g14610) in the reverse orientation.
AVL013	ATGTCTTCAGCTCAAGATCCAT	Aligns to nucleotides 1 to 22 of *AtSYP61* (At1g28490).
AVL014	GGTCAAGAAGACAAGAACGAA	Aligns to nucleotides 715 to 735 of *AtSYP61* (At1g28490) in the reverse orientation.
AVL015	ATGGCCTCCTCCGAGGACGTC	Aligns to nucleotides 1 to 21 of *mRFP1*.
AVL016	GGCGCCGGTGGAGTGGC	Aligns to nucleotides 659 to 675 of *mRFP1* in the reverse orientation.
AVL017	**GGGGACAAGTTTGTACAAAAAAGCAGGCTTC**ATGAATCGCATTTCAACCAG	Aligns to nucleotides 1 to 20 of *hopK1*. Adds an attB1 region (in bold; for Gateway® cloning) to the 5' end of the primer.

Name	Sequence 5' ⟶ 3'	Comments
AVL018	**GGGGACCACTTTGTACAAGAAAGCTGGGTC**GCAGTAGAGCGTGTCGCGAC	Aligns to nucleotides 995 to 1014 of *hopK1* in the reverse orientation. Adds an attB2 region (in bold; for Gateway® cloning) to the 5' end of the primer.
AVL019	**CATATG**ATCAGTTCGCGGATCGGCGGG	Aligns to nucleotides 1 to 24 of *hopM1* from *Pst* DC3000. Adds an NdeI restriction site (in bold, the last 3 nucleotides belong to the ATG start codon) to the 5' end of the primer.
AVL020	**GAATTC**TTATGCACCTTTCCAGCCACCCAC	Aligns to nucleotides 880 to 900 of *hopM1* from *Pst* DC3000 in the reverse orientation. Adds an EcoRI restriction site (in bold) and a stop codon to the 5' end of the primer.
AVL021	**AAAAGCAGGCTCCGAATTCGCCCTT**ATGAATTTTACTGGCTATTCTC	Sequence in bold aligns to pCR8 plasmid while the remainder aligns to nucleotides 1 to 22 of *AtPR1*.
AVL022	**ACGAACACTGCGAGGGACATGCCAT**ATCTTGAGCTTTCGAGGGAA	Sequence in bold aligns after the signal peptide of *VLRB* while the remainder aligns to nucleotides 65 to 84 of *AtPR1* (At2g14610). Aligns in the reverse orientation.
AVL023	**CCGATCCGCGAACTGATCAT**ATCTTGAGCTTTCGAGGGAA	Sequence in bold aligns to nucleotides 1 to 20 of *hopM1* while the remainder aligns to nucleotides 65 to 84 of *AtPR1*. Aligns in the reverse orientation.
AVL024	**TTCCCTCGAAAGCTCAAGAT**ATGATCAGTTCGCGGATCGG	Sequence in bold aligns to nucleotides 65 to 84 of *AtPR1* while the remainder aligns to nucleotides 1 to 20 of *hopM1* from *Pst* DC3000.

Name	Sequence 5' ⟶ 3'	Comments
AVL025	**TTCCCTCGAAAGCTCAAGAT**AT GGCATGTCCCTCGCAGTGTTC	Sequence in bold aligns to nucleotides 65 to 84 bp of *AtPR1*, while the remainder aligns after the signal peptide of *VLRB*, but adding before an ATG codon.
AVL026	TGGATCTTGAGCTGAAGACAT**G AATTCGCCAGAACCAGCAGCG GAGCCAGCGGATCC**CGTGGTC GTAGCAACGTAGC	The 5' end of the primer aligns to nucleotides 1 to 21 of *AtSYP61* while the 3' end of the primer aligns to the 3' end of *VLRB* before the stalk region. Adds a flexible linker (in bold) coding for Gly-Ser-Ala-Gly-Ser-Ala-Ala-Gly-Ser-Gly-Glu-Phe. Aligns in the reverse orientation.
AVL027	GCTACGTTGCTACGACCACG**GG ATCCGCTGGCTCCGCTGCTGGT TCTGGCGAATTC**ATGTCTTCAGC TCAAGATCCA	The 5' end of the primer aligns to the 3' end of *VLRB* before the stalk region while the 3' end of the primer aligns to nucleotides 1 to 21 of *AtSYP61*. Adds a flexible linker (in bold) coding for Gly-Ser-Ala-Gly-Ser-Ala-Ala-Gly-Ser-Gly-Glu-Phe.
